# Anatomic and Histological Features of the Extensor Digitorum Longus Tendon Insertion in the Proximal Nail Matrix of the Second Toe

**DOI:** 10.3390/diagnostics10030147

**Published:** 2020-03-07

**Authors:** Patricia Palomo-López, Marta Elena Losa-Iglesias, Ricardo Becerro-de-Bengoa-Vallejo, David Rodríguez-Sanz, Cesar Calvo-Lobo, Jorge Murillo-González, Daniel López-López

**Affiliations:** 1 University Center of Plasencia, Faculty of Podiatry, Universidad de Extremadura, 10600 Badajoz, Spain; patibiom@unex.es; 2 Faculty of Health Sciences, Universidad Rey Juan Carlos, 28922 Alcorcon, Spain; marta.losa@urjc.es; 3 School of Nursing, Physiotherapy and Podiatry, Universidad Complutense de Madrid, 28040 Madrid Spaindavidrodriguezsanz@ucm.es (D.R.-S.); 4 Department of Human, Anatomy and Embryology, Faculty of Medicine, Madrid Complutense University, 28040 Madrid, Spain; jmurillo@med.ucm.es; 5 Research, Health and Podiatry Group. Department of Health Sciences, Faculty of Nursing and Podiatry, Universidade da Coruña, 15403 Ferrol Spain; daniellopez@udc.es

**Keywords:** anatomy and histology, foot, nails, nail matrix, toe joint, tendons, toe phalanges, nail deformity, anatomic landmarks

## Abstract

Background: Anatomic and histological landmarks of the extensor digitorum longus (EDL) tendon insertion in the proximal nail matrix may be key aspects during surgery exposure in order to avoid permanent nail deformities. Objective: The main purpose was to determine the anatomic and histological features of the EDL’s insertion to the proximal nail matrix of the second toe. Methods: A sample of fifty second toes from fresh-frozen human cadavers was included in this study. Using X25-magnification, the proximal nail matrix limits and distal EDL tendon bony insertions were anatomically and histologically detailed. Results: The second toes’ EDLs were deeply located with respect to the nail matrix and extended superficially and dorsally to the distal phalanx in all human cadavers. The second toe distal nail matrix was not attached to the dorsal part of the distal phalanx base periosteum. Conclusions: The EDL is located plantar and directly underneath to the proximal nail matrix as well as dorsally to the bone. The proximal edge of the nail matrix and bed in human cadaver second toes are placed dorsally and overlap the distal EDL insertion. These anatomic and histological features should be used as reference landmarks during digital surgery and invasive procedures.

## 1. Introduction

Toe-tip disorders seem to be common and often affect the nail matrix and plate of the second toe [1]. Nail matrix and plate surgeries of the second toe distal phalanx may be performed secondary to trauma, infection, neoplasm, iatrogenic injuries, degenerative pathologies, and self-inflicted injuries [1,2].

Thus, surgeries for nail bed reconstruction may require a deep understanding of the anatomy and histology of the nail matrix as well as the relationship between the nail and surrounding tissues. The matrix may frequently develop injuries and deformities secondary to surgeries of the second toe distal phalanx and its nail plate. In addition, the nail matrix may be considered as the main origin of the nail plate. A higher potential risk is presented for scarring procedures in the proximal nail bed compared with the distal nail bed [3]. Finally, nail deformities may be produced secondary to the aforementioned conditions [4].

Anatomic and histological landmarks of the matrix proximal edge may be key aspects during surgery exposure in order to avoid permanent nail deformities. Several studies have evaluated the nail anatomy [4,5,6,7,8,9,10,11] as well as the flexor and extensor hallucis brevis and abductor and adductor hallucis tendon insertions of the hallux proximal phalanx base with surgical implications [12]. Indeed, the anatomic and histologic relationship between the proximal nail matrix and the distal tendons insertion has already been detailed for the distal extensor pollicis longus tendon of the fingernail matrix in the thumb [13], as well as the extensor hallucis longus tendon insertion into the proximal nail matrix of the great toe [14]. Nevertheless, to our knowledge, this anatomic and histologic relationship between the proximal nail matrix and the extensor digitorum longus (EDL) tendon insertion has not yet been described in the lesser toes. Thus, the purpose of this study was to determine the anatomic and histological features of the EDL insertion to the proximal nail matrix of the second toe.

## 2. Materials and Methods

### 2.1. Sample

A total sample of 50 2nd toes from fresh-frozen human cadavers, with an age older than 18 years old and without any history of digital traumas, was included for the present study. An age mean of 62.5 years with a range from 45 to 80 years old was obtained from the total sample, including 21 women and 29 men. These 2nd toes were obtained from 26 left feet and 24 right feet from 50 cadavers. Some toes were excluded due to the presence of digital deformities. The human cadavers proceeded from the Human Anatomy and Embriology I Department of the School of Medicine from the Complutense University of Madrid (Spain). This research was carried out according to The Strengthening the Reporting of Observational Studies in Epidemiology criteria [15].

### 2.2. Ethical Considerations

All experimental protocols were approved (1 March 2018) by the Ethical Committee of the Rey Juan Carlos University (Spain), with approval code 0801201800818. Furthermore, all methods were carried out according to the relevant regulations and guidelines. Consent for the present study was obtained from the Human Anatomy and Embriology I Department of the School of Medicine from the Complutense University of Madrid (Spain), which included informed consent as part of the cadaver donation process.

### 2.3. Procedure

A longitudinal incision of the dorsal skin was carried out along the shaft of the distal phalanx extending through the eponychium. In addition, two radial incisions were performed proximal to the junctions of the nail fold and nail walls [16,17]. Furthermore, skin flaps were retracted to expose the matrix dorsal part and the complete nail plate length.

**Figure 1:**
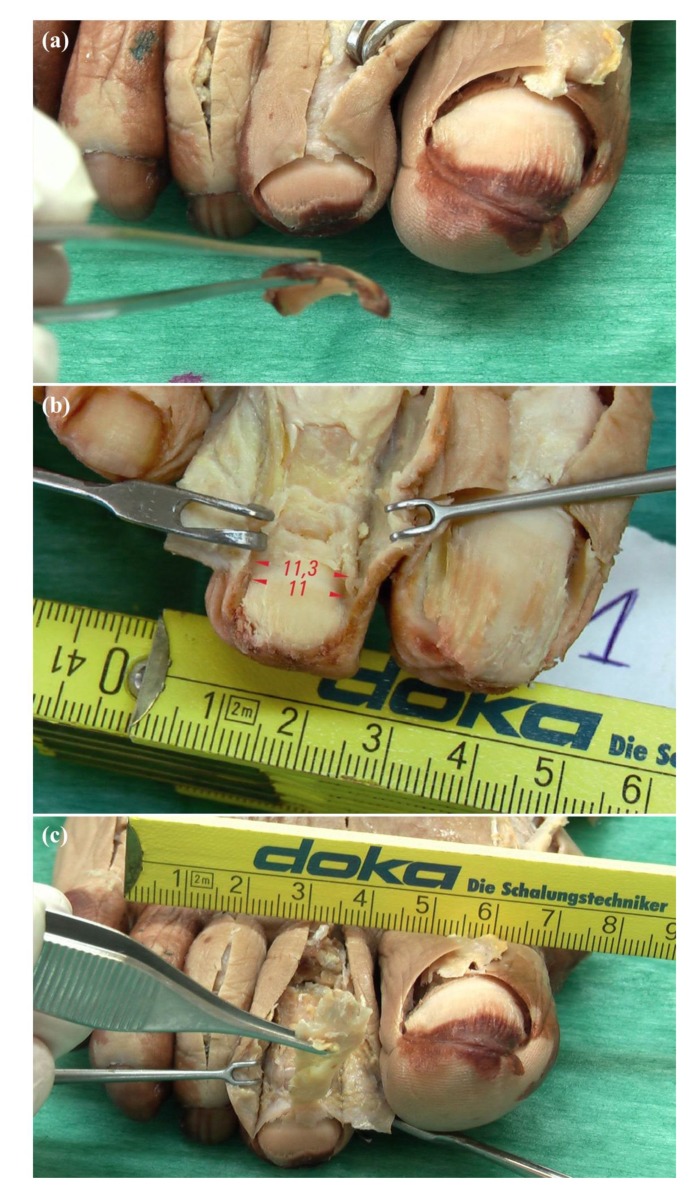
Anatomical study procedure: (**a**) The nail plate was gently elevated and sharply excised from side to side; (**b**) Color and texture of the nail matrix and bed (Red arrows and numbers showed the nail matrix width in mm at the proximal and distal nail parts); (**c**) Exposure of the distal insertion of the extensor digitorum longus (EDL) tendon at the base of distal phalanx until the proximal nail matrix fold was reached.

Toenails were removed atraumatically, preserving the nail bed and matrix as well as the eponychium. These procedures were achieved by firstly loosening the nail plate from the nail matrix using a fine elevator. Nail plates were elevated and sharply excised from side to side. Nail plate removal enabled the accurate inspection of the nail matrix borders (Figure 1a).

Proximal nail matrix edges were determined by visual inspection of the proximal origin location of the nail plate. Proximal nail plate origins were delimited as the proximal matrix edges. Afterwards, the color and texture of the nail matrix and bed were observed. Indeed, the nail matrix presented a white color, while nail beds presented a red color (Figure 1b).

Proximal matrix edges were identified with respect to the distal limits of the EDL tendon at the distal bony insertion by both X25-magnification optical microscope and visual inspection (Figure 1c).

Distal EDL tendon insertions were landmarked by visual inspection and confirmed by histological cross-sections analyses in the pathology laboratory. Standard longitudinal sections were stained by Tetrachrome VOF-III GS stains (light green SF/or fast green FCF-methyl blue-Orange G-acid fuchsin).

### 2.4. Outcome Measurements

Nail matrix proximal edges were stated in all cadavers. Distal EDL insertions were clearly identified in all cadavers. Thus, anatomical landmarks were macroscopically measured and delimited, as described below, according to the following variables. Variable-1: Matrix width measurements were carried out from the lateral to the medial side at the most proximal part of the 2nd toe. Variable-2: The same measurements were carried out at the distal aspect of the matrix, where colors changed with the nail bed. Variable-3: Matrix lengths were determined from proximal to distal sides along the shaft of the 2nd toe. Variable-4: Nail bed lengths were measured as above from where the color changed to the bed end. Variable-5: EDL tendon widths were determined from the lateral to the medial side at the distal interphalangeal joint line.

### 2.5. Statistical Analysis

Outcome measurements were detailed as the mean, standard deviation (SD), upper and lower limits of the 95% confidence interval (95% CI) and minimum and maximum values, as well as the median. All analyses were carried out with SPSS 19.0 software (Chicago, IL, USA). Data normality was assessed by the Kolmogorov–Smirnov test. In order to compare outcome measurements for sex and lower limb side distributions, the Student *t* test for independent samples and the Mann–Whitney U test were used for parametric and non-parametric data, respectively.

## 3. Results

The dimensions of the matrix, bed and EDL tendon of the second toes (*n* = 50) are presented in Table 1. Nail matrix widths were larger at the distal nail parts (8.80 ± 1.86 mm) than the proximal nail parts (8.07 ± 1.53 mm). Nail bed lengths (5.68 ± 1.40 mm) were larger than nail matrix lengths (2.37 ± 0.29 mm). In addition, the EDL tendon widths showed a mean ± SD of 2.49 ± 0.66 mm at the distal interphalangeal joint line.

According to sex comparisons presented in Table 2, statistically significant differences (*p* < 0.001) were shown for the nail matrix width at the proximal nail part showing a larger width for men compared to women. The rest of the outcome measurements did not show any statistically significant differences (*p* > 0.05) for sex distribution.

Regarding lower limb side comparisons presented in Table 3, there were no statistically significant differences (*p* > 0.05) between left and right sides for any outcome measurement.

This study analyzed the functional link of the nail to the second toe distal phalanx and various distal interphalangeal joint structures, such as EDL tendon fibers and collateral ligaments. EDL tendons continued from their bony insertions overlapping the distal phalanges, and the collateral ligaments formed an integrated network on both joint sides, thereby helping to anchor nail margins.

**Figure 2:**
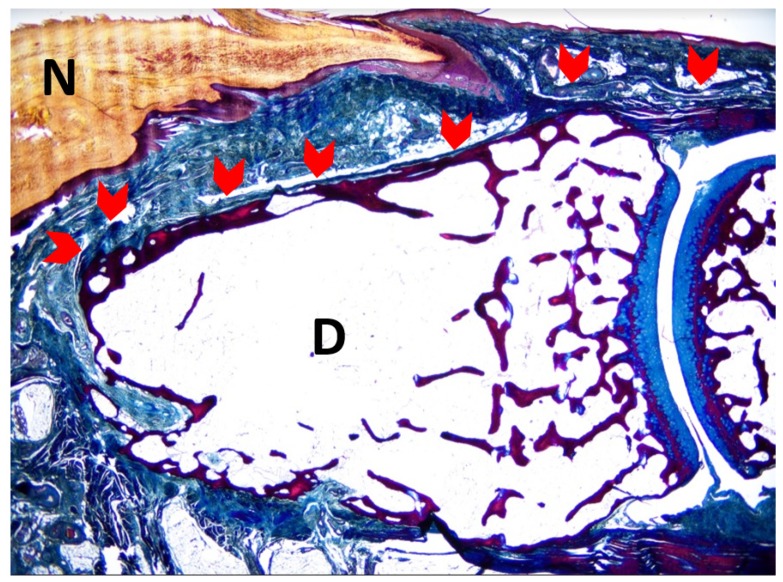
Sagittal section of the second toe at the midpoint of the nail matrix. The superficial fibers of the Extensor Digitorum Longus (EDL) tendon bundles extend to the dorsal aspect of the distal phalanx of the second toe (red arrowheads). N—nail plate; D—distal phalanx. (5× magnification). Tetrachrome VOF-III GS stain (light green SF/or fast green FCF, methyl blue, Orange G, and acid fuchsin). Scale bar = 5× magnification

According to the findings obtained in our study, we have verified, through dissection and histologic studies, that EDL tendons ended at the dorsal area of the second toe distal phalanx and were attached to the complete phalanx, ending at the nail (Figure 2).

**Figure 3:**
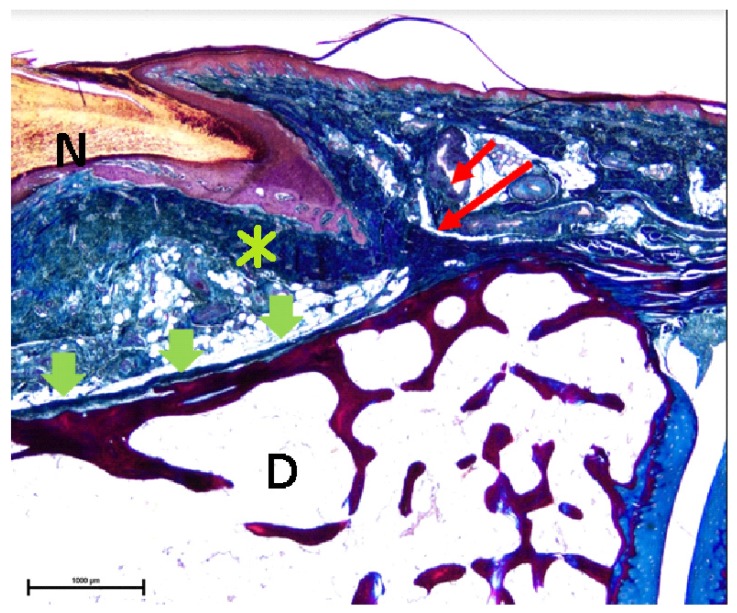
High-power magnification detail of the superficial fibers of the Extensor Digitorum Longus (EDL) bundles (red arrows) attached at the base of ventral matrix (asterisk) to the underlying distal phalanx (D) bone, crossing the deep fibers of the EDL tendon bundles (green arrowheads). Superficial fibers of the EDL tendon expansion to the eponychium (red arrow), which run distally along the complete dorsal aspect of the distal phalanx until its end (20× magnification). Tetrachrome VOF-III GS stain (light green SF/or fast green FCF, methyl blue, Orange G, and acid). N—nail plate; D—distal phalanx. Scale bar = 20× magnification

Indeed, there was a close relationship between EDL tendons and the matrix. EDL superficial fibers were attached to the plantar matrix base underlying the distal phalanx (DF) bone and crossing the EDL deep fibers. EDL superficial fibers were expanded to the eponychium (Figure 3), which ran distally along the complete dorsal region of the distal phalanx.

## 4. Discussion

Nails may be considered as specialized parts of the skin, such as an “epidermal appendage”. Indeed, nails seemed to be functionally integrated with the musculoskeletal system. The second toe may play a key role in both morphological and biomechanical foot functions. At standing position, second toes showed more pressure than the fifth metatarsal heads and heels [2]. In addition, great toes produced a pressure about twice of the total pressure of the other four toes. Nevertheless, second toes supported the largest pressure after the hallux. During walking activities, as great toes and second toes were passively dorsiflexed, foot longitudinal arches were raised, the rearfoot was supinated, legs were externally rotated, and plantar aponeurosis was tensed [18]. Great toes and second toe disorders could modify subjects’ static and dynamic balance, as well as gait, stance, and the entire erect bipedal locomotion process.

Toenails may play a key functional role regarding proprioceptive inputs, protective features, and control of toe pulps. Toenails seem to be related to the matrix of the distal phalanx periosteum. Matrix damage may lead to nail plate alterations [19].

The matrix location with respect to the EDL tendon may present clinical implications. Several studies have detailed the close relationship between the EDL tendon and the nail matrix, although prior studies have not delimited the exact anatomic distance in the foot [5,6,8,9,10,11,13,20]. These studies have been carried out in the hand and thus there are a lack of landmark studies in the foot region. Authors have not found a similar study that quantifies the distances between the nail matrix and the EDL tendon insertion in the second toes.

A study of the matrix surface anatomy of the finger nail carried out by Reardon et al. [10]. delimited the distance from the nail bed proximal edge with respect to the distal interphalangeal joint. While these investigators showed that the extensor tendon insertions were always more proximal to the most proximal part of the matrix, no attempts were performed to quantify this distance. Regarding ingrown nails, a similar research work examined the matrix extent of toenails and reported that the matrix was extended to the extensor tendon insertion, although the distance was not also quantified [11]. Thus, our anatomic and histological study demonstrated the proximal edge of the matrix in the human cadavers second toes and the distal EDL tendon bony insertions.

A study limitation was that the nail plate proximal origin was delimited as the nail matrix proximal limit in order to determine its distal length, and thus the matrix was not evaluated more proximally than the nail plate. This could be a possible limitation, as the nail matrix may be extending more proximally than the nail plate itself. According to the sagittal section of the second toe at the midpoint of the nail matrix (Figure 2; Figure 3), often the nail matrix may present lateral matrix horns which may be extended as far proximally as the 75% of the distance to the distal interphalangeal joint crease [21]. Further research studies are needed in order to establish more accurate measurements. Furthermore, the impact of formalin preservation and histological process could have influenced the tissue relationships and dimensions. Although formalin retained shape and size of organs and vessels, Genelyn and Imperial College London soft-preservation (ICL-SP) solution technique may faithfully mimic cadavers’ joints compared to un-embalmed cadavers [22]. Thus, future studies should compare our findings using different embalming solutions on the human cadavers’ tissues. Finally, a main limitation was that our study was only a descriptive study according to our aim, which was to detail the anatomic and histological features of the EDL insertion to the proximal nail matrix of the second toe. Therefore, future studies should compare differences among outcome measurements such as the nail matrix width at the proximal nail part, the nail matrix width at the distal nail part, the length of the matrix, the length of the bed, and the tendon width. Several debilitating dermatoses may affect the nail unit, including papulosquamous disease and depigmented skin conditions.

## 5. Conclusions

In conclusion, the nail matrix and bed proximal edges of human cadavers’ second toes were positioned dorsally and overlapped the distal EDL tendon until its distal bony insertion, ending at the nail in all human cadavers’ feet. EDL tendons were not directly connected to the nail matrix due to the nail bed corium and nailbed mesenchyme being located between the matrix or nailbed epithelium and EDL. These anatomic and histological features should be used as reference landmarks during digital surgery and invasive procedures.

## Figures and Tables

**Table 1:** Dimensions of the matrix, bed and EDL tendon of the second toes (*n* = 50).

Outcome Measurements (mm; *n* = 50)	Mean ± SD	95% CI (LL-UL)	Median	Minimum	Maximum
**1.** Nail matrix width at the proximal nail part (mm)	8.07 ± 1.53	(8.54–9.39)	8.00	5.50	11.30
**2.** Nail matrix width at the distal nail part (mm)	8.80 ± 1.86	(8.28–9.31)	8.40	5.80	12.00
**3.** Length of matrix (mm)	2.37 ± 0.29	(2.28–2.45)	2.40	1.60	2.80
**4.** Length of bed (mm)	5.68 ± 1.40	(5.26–6.06)	5.40	3.30	8.70
**5** Tendon width (mm)	2.49 ± 0.66	(2.30–2.68)	2.60	1.50	4.0

Abbreviations: CI, confidence interval; EDL, extensor digitorum longus; mm, millimeters; LL, lower limit; UL, upper limit.

**Table 2:** Sex differences for dimensions of the matrix, bed and EDL tendon of the second toes (*n* = 50).

Outcome Measurements (mm)	Men (*n* = 29) Mean ± SD (Range)	Women (*n* = 21) Mean ± SD (Range)	*p*-Value
**1.** Nail matrix width at the proximal nail part (mm)	9.02 ± 1.09 (8.00–11.30)	6.77 ± 1.02 (5.50–8.20)	<0.001 ^†^
**2.** Nail matrix width at the distal nail part (mm)	8.70 ± 1.90 (5.90–12.00)	8.92 ± 1.85 (5.80–11.80)	0.683 *
**3.** Length of matrix (mm)	2.39 ± 0.31 (1.80–2.80)	2.34 ± 0.28 (1.60–2.80)	0.535 *
**4.** Length of bed (mm)	5.67 ± 1.48 (3.90–8.70)	5.69 ± 1.31 (3.30–8.50)	0.555 ^†^
**5** Tendon width (mm)	2.41 ± 0.64 (1.50–3.50)	2.60 ± 0.68 (1.60–4.00)	0.215 ^†^

Abbreviations: EDL, extensor digitorum longus; mm, millimeters. * Student *t* test for independent samples was used. † Mann–Whitney *U* test was used. For all analyses, statistically significant differences were set at *p* < 0.05.

**Table 3:** Lower limb side differences for dimensions of the matrix, bed and EDL tendon of the second toes (*n* = 50).

Outcome Measurements (mm)	Left (*n* = 26) Mean ± SD (Range)	Right (*n* = 24) Mean ± SD (Range)	*p*-Value
**1.** Nail matrix width at the proximal nail part (mm)	8.21 ± 1.60 (5.70–11.30)	7.92 ± 1.48 (5.50–10.90)	0.785 ^†^
**2.** Nail matrix width at the distal nail part (mm)	8.58 ± 1.70 (5.90–11.80)	9.03 ± 2.04 (5.80–12.00)	0.393 *
**3.** Length of matrix (mm)	2.42 ± 0.26 (1.90–2.80)	2.31 ± 0.32 (1.60–2.80)	0.194 *
**4.** Length of bed (mm)	5.87 ± 1.49 (3.30–8.50)	5.47 ± 1.29 (3.90–8.70)	0.398 ^†^
**5** Tendon width (mm)	2.60 ± 0.64 (1.60–4.00)	2.38 ± 0.67 (1.50–3.50)	0.398 ^†^

Abbreviations: EDL, extensor digitorum longus; mm, millimeters. * Student *t* test for independent samples was used. † Mann–Whitney *U* test was used. For all analyses, statistically significant differences were set at *p* < 0.05.

## Data Availability

Data will be available upon request to the corresponding author.
